# Local tirofiban infusion for remnant stenosis in large vessel occlusion: tirofiban ASSIST study

**DOI:** 10.1186/s12883-020-01864-4

**Published:** 2020-07-20

**Authors:** Yong-Won Kim, Sung-Il Sohn, Joonsang Yoo, Jeong-Ho Hong, Chang-Hyun Kim, Dong-Hun Kang, Yong-Sun Kim, Seong-Joon Lee, Ji Man Hong, Jin Wook Choi, Yang-Ha Hwang, Jin Soo Lee

**Affiliations:** 1 Department of Neurology, School of Medicine, Kyungpook National University, 130 Dongdeok-ro, Jung-gu, Daegu, 41944 South Korea; 2 Department of Neurology, Keimyung University School of Medicine, Daegu, South Korea; 3 Department of Neurology, National Health Insurance Service Ilsan Hospital, Goyang, South Korea; 4 Department of Neurosurgery, Keimyung University School of Medicine, Daegu, South Korea; 5 Department of Neurosurgery, School of Medicine, Kyungpook National University, Daegu, South Korea; 6 Department of Radiology, School of Medicine, Kyungpook National University, Daegu, South Korea; 7 Department of Neurology, Ajou University Medical Center, Ajou University School of Medicine, 164 World cup-ro, Yeongtong-gu, Suwon, 16499 South Korea; 8 Department of Radiology, Ajou University School of Medicine, Suwon, South Korea

**Keywords:** Ischemic stroke, Intracranial atherosclerosis, Thrombectomy

## Abstract

**Background:**

Compared with embolic occlusions, intracranial atherosclerotic stenosis (ICAS)-related large vessel occlusions (LVOs) often require rescue treatment following mechanical thrombectomy (MT). Herein, we hypothesized that local tirofiban infusion can be effective and safe for remnant stenosis in LVO during endovascular treatment and can improve clinical outcomes.

**Methods:**

This observational multicenter registry study (January 2011 to February 2016) included patients with ICAS who underwent endovascular treatment for LVO within 24 h after stroke onset. An underlying fixed focal stenosis at the occlusion site observed on cerebral angiography during and after MT was retrospectively determined as a surrogate marker of ICAS. Procedural and clinical outcomes were compared between the tirofiban and non-tirofiban groups.

**Results:**

Of 118 patients, 59 received local tirofiban infusion. Compared to the non-tirofiban group, patients were older (non-tirofiban group versus tirofiban group; median, 63 years vs. 71 years, *p* = 0.015) and the onset-to-puncture time was longer (median, 275 min vs. 395 min, *p* = 0.036) in the tirofiban group. The median percent of residual stenosis prior to rescue treatment tended to be higher in the tirofiban group (80 [71–86] vs. 83 [79–90], *p* = 0.056). Final reperfusion success (modified Treatment In Cerebral Ischemic 2b–3) was more frequent (42.4%vs. 86.4%, *p* = 0.016) and post-procedure parenchymal hematoma type 2 and/or thick subarachnoid hemorrhages were less frequent (15.3%vs. 5.1%, *p* = 0.068) in the tirofiban group. The frequency of favorable outcomes 3 months after endovascular treatment (modified Rankin Scale 0–2) was significantly higher in the tirofiban group (32.2% vs. 52.5%, *p* = 0.025), and tirofiban administration was an independent predictor of favorable outcomes (odds ratio, 2.991; 95% confidence interval, 1.011–8.848; *p* = 0.048).

**Conclusions:**

Local tirofiban infusion can be a feasible adjuvant treatment option for patients with ICAS-LVO.

## Background

Since randomized controlled trials for mechanical thrombectomy (MT) were successful, endovascular revascularization therapy (ERT) has been established as a standard treatment for acute ischemic stroke (AIS) with large vessel occlusion (LVO) of the intracranial anterior circulation [1–5]. MT is mostly based on stent retrieval or contact aspiration, which are designed for removing embolic clots in the occlusion vessel. However, if the occlusion is caused by intracranial atherosclerotic stenosis (ICAS), these MT methods may not be sufficient for recanalization and reperfusion, and rescue treatment is frequently required following MT [6–11]. Until now, angiographic and clinical outcomes of ERT for ICAS-LVO have been reported to be challenging [12–14].

ICAS is a common cause of stroke, especially in Asian populations [15, 16]. In situ thrombosis (IST) is a major mechanism involved in emergent ICAS-LVO [17, 18]. In addition, the endothelium of the ICAS can be injured by MT [19, 20]. This thrombogenic milieu can cause thrombus propagation or reocclusion even after partial recanalization [6, 9, 21, 22]. Therefore, stabilization of thrombogenic lesions should be considered for ICAS-related LVO.

In the current Tirofiban for Acute Serious Stroke Due to Intracranial in situ Thrombosis (Tirofiban ASSIST) study, we hypothesized that tirofiban, a locally infused antiplatelet agent, would stabilize the thrombogenic lesion in ICAS-LVO and improve clinical and angiographic outcomes. Therefore, we aimed to evaluate the safety and efficacy of intra-arterial tirofiban administration during ERT and to identify if this treatment is a predictor of favorable clinical outcomes in ICAS-LVO.

## Methods

### Patients

In this retrospective case–control study, the patients were recruited from the Acute Stroke due to Intracranial Atherosclerotic occlusion and Neurointervention Korean Retrospective (ASIAN KR) registry, which included databases from three stroke centers in Korea (from January 2011 to February 2016) [23]. Before data integration, all ASIAN KR data were de-identified. The criteria for inclusion were as follows: (1) patients had acute occlusion of the intracranial internal carotid artery (ICA), middle cerebral artery (MCA) M1, MCA M2, and vertebrobasilar artery; (2) the time from symptom onset to groin puncture was within 24 h; and (3) patients were diagnosed with ICAS-LVO, which was retrospectively evaluated on the cerebral angiography as the etiology of stroke.

Patients were excluded if (1) the extracranial target arterial occlusion and/or tandem intracranial large arterial occlusion was present, (2) there were undetermined angiographic etiologies because the occlusion was never recanalized during primary MT, or (3) if patients had other etiologies of stroke, including vasculitis, arterial dissection, or Moyamoya disease.

The institutional review board in each center approved this study. The requirement for informed consent was waived because of the retrospective nature of this study. Intra-arterial tirofiban in Korea has been used for ERT with approval from the Korean Food and Drug Administration for each institution.

### Etiologic classification of target arterial lesion

The etiology of target arterial lesion was classified based on angiographic findings during ERT. If there was no residual stenosis on angiography after reperfusion, the etiology was classified as an embolism [9, 22]. In contrast, ICAS was defined using the following conditions: (1) presence of residual stenosis over 70% and (2) reocclusion tendency or flow impairment with residual stenosis less than 70% [9, 22]. If recanalization was not achieved throughout the ERT or without angioplasty or stenting, it was classified as intractable. The etiologic classification was performed by two experienced stroke neurologists, and a consensus was reached (Y.H.H. and J.S.L.).

### Endovascular procedures

Stent retrieval and contact aspiration were mainly performed as primary MT strategies. If successful reperfusion was achieved but remnant ICAS was seen, follow-up angiography was performed 10–30 min after reperfusion. If the stenosis was aggravated, distal flow stagnation developed, or reocclusion occurred, repetitive MT or other rescue treatments, including switching MT strategy, intracranial tirofiban infusion, and balloon angioplasty and/or stenting, were applied. The decision of rescue treatment strategies was based on the neurointerventionists’ discretion.

Patients in the tirofiban group were locally administered with 0.5 mg to 2.0 mg of tirofiban as a rescue treatment. Additionally, 0.5 mg (2 ml) of tirofiban was diluted with 8 ml of normal saline or 1 mg (4 ml) of tirofiban with 6 ml of normal saline for intra-arterial local infusion, and the 10 ml of diluted tirofiban was manually administered approximately at a rate of 1 ml/min [6].

### Clinical and angiographic data

We analyzed the clinical and demographic data of the patients, including National Institute of Health Stroke Scale (NIHSS) scores, Alberta Stroke Program Early CT Scores (ASPECTS), and pre-stroke modified Rankin Scale (mRS) scores at admission. The Arterial Occlusive Lesion (AOL) grade was used for the measurement of recanalization in the target arterial lesion, and AOL grade 2–3 was considered an indicator of successful recanalization [24]. The degree of remnant stenosis prior to rescue treatment was estimated by the Warfarin-Aspirin Symptomatic Intracranial Disease method [25]. Successful reperfusion was defined as a modified Treatment In Cerebral Ischemia (mTICI) score of 2b or 3 based on the final angiography [24]. Brain CT was performed immediately and 12–24 h after ERT to evaluate hemorrhagic complications. Intracranial hemorrhages were classified based on the European Cooperative Acute Stroke Study [26]. Subarachnoid hemorrhage (SAH) severity was graded according to the modified Fisher scale [27]. Serious hemorrhagic complications were defined as parenchymal hematoma type 2 and/or a thick SAH with or without intraventricular hemorrhage (modified Fisher grade 3 or 4 of SAH). Postprocedural final infarct volume was measured by diffusion-weighted imaging (J.W.C.) using NordicICE semi-automated software (NordicNeuroLab, Bergen, Norway). Clinical outcomes were evaluated with mRS at 3 months after ERT. The mRS score was assessed by a certified neurologist or research nurse in each center during outpatient visit at 3 months after ERT. For patients who were unable to visit the outpatient department, structured telephone interview with the patient or family was conducted. A favorable clinical outcome was defined as an mRS score of ≤2 or no change compared with the premorbid mRS.

### Statistics

Chi-square tests or Fisher’s exact tests were used for categorical variables. Mann–Whitney U tests were used for continuous variables. A binary logistic regression analysis was performed to identify whether local tirofiban administration was an independent predictor of favorable clinical outcomes at 3 months and serious hemorrhagic complications. Age, sex, balloon angioplasty and/or stenting, and variables with *p* < 0.20 in the univariate analysis were included in the binary logistic regression analysis for favorable clinical outcomes at 3 months. For serious hemorrhagic complications, onset-to-reperfusion time and variables with *p* < 0.20 in the univariate analysis were included in the binary logistic regression analysis. For all analyses, *p* < 0.05 was considered statistically significant. Statistical analyses were performed using SPSS 22.0 (IBM, Armonk, NY).

## Results

### Demographics and baseline characteristics

**Fig. 1:**
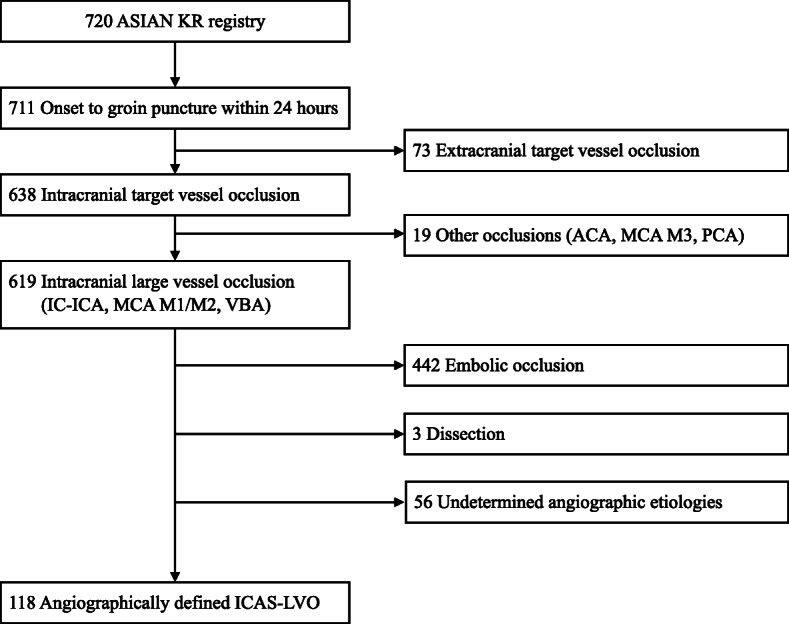
Flowchart of this study. ACA, anterior cerebral artery; ASIAN KR, Acute Stroke due to Intracranial Atherosclerotic occlusion and Neurointervention Korean Retrospective; ICAS, intracranial atherosclerotic stenosis; IC-ICA, intracranial internal carotid artery; LVO, large vessel occlusion; MCA, middle cerebral artery; VBA, vertebrobasilar artery

A total of 119 patients were included in this study (Fig. 1). Among them, 59 patients received local tirofiban infusion as a rescue treatment. Baseline characteristics and stroke risk factors are compared in Table 1. The median age of the patients was higher in the tirofiban group than in the non-tirofiban group (non-tirofiban group versus tirofiban group; 63 [55–75] versus 71 [61–78], *p* = 0.015). The median initial NIHSS scores (15 [12–21] versus 14 [10–20], *p* = 0.322) and the median ASPECTS scores (8 [4.5–9.5] vs. 8 [6–9], *p* = 0.530) did not significantly differ between the two groups. Further, the use of intravenous recombinant tissue plasminogen activator (rtPA) did not significantly differ between the two groups (49.2% versus 33.9%, *p* = 0.093). Although the incidence of dyslipidemia was higher in the non-tirofiban group than in the tirofiban group (42.4% versus 23.7%, *p* = 0.031), other risk factors of stroke did not significantly differ between the two groups.

**Table 1:** Comparison of the baseline characteristics of the patients in the tirofiban and non-tirofiban groups

	Non-tirofiban group (*n* = 59)	Tirofiban group (*n* = 59)	*P* value
Age, median (IQR)	63 (55–75)	71 (61–78)	0.015
Female	16 (27.1%)	23 (39.0%)	0.171
Prestroke mRS, median (IQR)	0 (0–0)	0 (0–0)	0.438
Initial NIHSS, median (IQR)	15 (12–21)	14 (10–20)	0.322
ASPECTS, median (IQR)	8 (4.5–9.5) (*n* = 41)	8 (6–9) (*n* = 46)	0.530
Intravenous rtPA	29 (49.2%)	30 (33.9%)	0.093
Target occlusion location			0.766
Terminal ICA	9 (15.3%)	8 (13.6%)	
MCA M1	34 (57.6%)	38 (64.4%)	
MCA M2	2 (3.4%)	3 (5.1%)	
VBA	14 (23.7%)	10 (16.9%)	
Hypertension	38 (64.4%)	38 (64.4%)	> 0.999
Diabetes mellitus	19 (32.2%)	18 (30.5%)	0.843
Dyslipidemia	25 (42.4%)	14 (23.7%)	0.031
Atrial fibrillation	12 (20.3%)	9 (15.3%)	0.470
Coronary disease	3 (5.1%)	4 (6.8%)	> 0.999^a^
Smoking	21 (35.6%)	23 (39.0%)	0.703
Prior antiplatelet	6 (10.2%)	13 (22.0%)	0.080
Prior anticoagulant	4 (6.8%)	1 (1.7%)	0.364^a^

*ASPECTS* Alberta Stroke Program Early CT Score, *ICA* internal carotid artery, *IQR* interquartile range, *MCA* middle cerebral artery, *mRS* modified Rankin Scale, *NIHSS* National Institute of Health Stroke Scale, *rtPA* recombinant tissue plasminogen activator, *VBA* vertebro-basilar artery

^a^Fisher’s exact t-test

### Comparisons of angiographic data and outcomes

The procedural, angiographic, and clinical outcomes for each group are summarized in Table 2. The median time from stroke symptom onset to groin puncture was shorter in the non-tirofiban group than in the tirofiban group (275 min versus 395 min, *p* = 0.036). The rate of aspiration thrombectomy and stent retriever thrombectomy, which were used as primary MT strategies, was similar in both groups. Compared to the non-tirofiban group, the median percent of remnant stenosis prior to rescue treatment tended to be higher in the tirofiban group (80 [71–86] versus 83 [79–90], *p* = 0.056). Additional rescue treatments such as thrombectomy device switching, balloon angioplasty, and/or stenting were used more frequently in the non-tirofiban group than in the tirofiban group. However, no significant difference was found between the two groups with respect to the use of these treatments, with the exception of intracranial balloon angioplasty. No differences were noted in the rate of successful recanalization graded by AOL between the two groups (69.5% versus 69.5%, *p* > 0.999); however, the rate of successful reperfusion graded by mTICI was higher in the tirofiban group than in the non-tirofiban group (42.4% versus 86.4%, *p* = 0.016). Additionally, the incidence of SAH (*p* = 0.027) and intraventricular hemorrhage (*p* = 0.032) was higher in the non-tirofiban group than in the tirofiban group, but the occurrence of intracerebral hemorrhage did not differ between the groups (*p* = 0.311). The final infarct volume after ERT was smaller in the tirofiban group than in the non-tirofiban group (38.8 ml versus 18.5 ml, *p* = 0.023).

**Table 2:** Details of the endovascular treatment and clinical outcomes

	Non-tirofiban group (*n* = 59)	Tirofiban group (*n* = 59)	*P* value
Onset-to-puncture time	275 (210–482)	395 (274–580)	0.036
Puncture-to-final angiography time	81 (60–101)	65 (42–108)	0.064
Onset-to-reperfusion time	380 (298–646)	467 (345–675)	0.066
First-line endovascular treatment			0.096
Aspiration thrombectomy	29 (49.2%)	33 (55.9%)	
Stent retriever	23 (39.0%)	25 (42.4%)	
Local fibrinolytics	1 (1.7%)	1 (1.7%)	
Angioplasty	6 (10.2%)	0	
Immediate reocclusion after first endovascular method	10 (17.9%)	24 (41.4%)	0.006
Degree of residual stenosis prior to rescue treatment (%)	80 (71–86)	83 (79–90)	0.056
Rescue treatments			
Local tirofiban infusion only	0	48 (81.4%)	< 0.001
Stent retriever to aspiration	1 (1.7%)	0	> 0.999^a^
Aspiration to stent retriever	8 (13.6%)	3 (5.1%)	0.113
Intracranial balloon angioplasty	9 (15.3%)	2 (3.4%)	0.027
Intracranial stenting	12 (20.3%)	6 (10.2%)	0.124
Final AOL 2–3	41 (69.5%)	41 (69.5%)	> 0.999
Final mTICI 2b–3	25 (42.4%)	51 (86.4%)	0.016
Postprocedural reocclusion	12 (37.5%, *n* = 32)	2 (4.4%, *n* = 45)	< 0.001
Intracerebral hemorrhage			0.311
HT type 1	4 (6.8%)	2 (3.4%)	
HT type 2	6 (10.2%)	3 (5.1%)	
PH type 1	3 (5.1%)	1 (1.7%)	
PH type 2	6 (10.2%)	3 (5.1%)	
Subarachnoid hemorrhage	6 (10.2%)	0	0.027^a^
Intraventricular hemorrhage	8 (13.6%)	1 (1.7%)	0.032^a^
Serious hemorrhagic complication^b^	9 (15.3%)	3 (5.1%)	0.068
Final infarct volume, *ml* (median, IQR)	38.8 (14.3–92.7)	18.5 (7.9–37.2)	0.023
mRS 0–2 at 3 months	19 (32.2%)	31 (52.5%)	0.025
Mortality	12 (20.3%)	4 (6.8%)	0.031

*AOL* arterial occlusive lesion, *ERT* endovascular revascularization therapy, *HT* hemorrhagic transformation, *mRS* modified Rankin Scale, *MT* mechanical thrombectomy, *mTICI* modified treatment in cerebral ischemia, *PH* parenchymal hematoma

^a^Fisher’s exact t-test; ^b^Serious hemorrhagic complications consist of parenchymal hematoma type 2 and/or subarachnoid hemorrhage Fisher grade 3–4

Repeat angiographies during admission after ERT were obtained in 32 patients in the non-tirofiban group and in 45 in the tirofiban group. The incidence of postprocedural reocclusion was significantly higher in the non-tirofiban group than in the tirofiban group (37.5% versus 4.4%, *p* < 0.001). A favorable outcome 3 months after ERT was more frequent in the tirofiban group than in the non-tirofiban group (32.2% versus 52.5%, *p* = 0.025).

Using a logistic regression model, local tirofiban infusion (*p* = 0.048) was found to be an independent predictor of favorable clinical outcomes (Table 3). In another regression model, local tirofiban infusion was not associated with serious hemorrhagic complications; however, the final infarct volume (*p* = 0.033) was independently associated with serious hemorrhagic complications (Table 4). Additionally, no significant interaction was found between tirofiban infusion and final infarct volume for serious hemorrhagic complications (*p* = 0.339).

**Table 3:** Binary logistic regression analysis for favorable clinical outcomes

Variables	Odds ratio (95% CI)	*p* value
Age	0.920 (0.871–0.971)	0.002
Female	0.616 (0.205–1.849)	0.388
Baseline NIHSS	0.851 (0.772–0.939)	0.001
Occlusion location		0.269
Terminal ICA	Ref.	
MCA M1	5.109 (0.911–28.663)	0.064
MCA M2	3.397 (0.239–48.368)	0.268
VBA	2.775 (0.387–19.921)	0.310
Onset to puncture time	1.000 (0.999–1.001)	0.818
Puncture to final reperfusion time	0.990 (0.978–1.002)	0.112
Successful reperfusion	1.986 (0.552–7.149)	0.261
Rescue balloon angioplasty and/or stenting	0.288 (0.072–1.157)	0.079
Local tirofiban infusion	2.991 (1.011–8.848)	0.048

*ICA* internal carotid artery, *MCA* middle cerebral artery, *NIHSS* National Institute of Health Stroke Scale, *VBA* vertebro-basilar artery

**Table 4:** Binary logistic regression analysis for serious hemorrhagic complications

Variables	Odds ratio (95% CI)	*p* value
Age	0.965 (0.894–1.042)	0.361
Intravenous rtPA	0.228 (0.019–2.804)	0.248
Prior use of oral antiplatelet or anticoagulant	1.624 (0.133–19.880)	0.705
Onset to final reperfusion time	0.999 (0.994–1.003)	0.620
Local tirofiban	1.362 (0.123–15.102)	0.801
Final infarct volume	1.010 (1.001–1.019)	0.033

*ERT* endovascular revascularization therapy, *rtPA* recombinant tissue plasminogen activator

## Discussion

In this study, we evaluated the safety and efficacy of local tirofiban infusion as a rescue ERT strategy for AIS for patients with ICAS-LVO. The main findings of this study were as follows: (1) the rates of successful reperfusion and favorable outcomes were higher in the tirofiban group than in the non-tirofiban group, and (2) despite its lytic characteristics, whereas the rate of hemorrhagic complications appeared to be the result of the final large infarct volume, it was lower in the tirofiban group than in the non-tirofiban group. Overall, results from this retrospective registry study suggested that local tirofiban infusion could be a safe and effective rescue treatment for patients with ICAS-LVO.

ICAS is a major etiology of LVO, especially in Asian populations, and is still challenging to manage during modern MT [12, 17, 22]. ICAS-related LVO may result from IST beyond a preexisting stenosis [6, 21, 22, 28]. In IST, the rupture of preexisting atherosclerotic plaques and the release of tissue factors from the endothelial surface can lead to a thrombogenic and platelet aggravating environment [18]. In addition, usual MT may induce plaque rupture and cause extensive arterial injury from the endothelium to the tunica media [19, 20]. Therefore, local thrombogenic conditions may be exacerbated, and this often causes the vessel to become reoccluded even after successful reperfusion is achieved by usual MT. Based on these data, early stabilization of the endothelium and intracranial atherosclerotic plaque is an important goal, and antiplatelet administration is ideal to stabilize the thrombogenic lesion. Since the underlying ICAS is hidden in LVO, pretreatment with oral antiplatelet agents cannot be applied in most cases; thus, infusible antiplatelet has been anecdotally used in the IST lesion as rescue treatment for intracranial LVO [6, 29, 30]. To this end, the glycoprotein IIb/IIIa inhibitor may play a crucial role in the prevention of fibrinogen-induced platelet aggregation and local thrombus formation [31].

Tirofiban is an infusible antiplatelet glycoprotein IIb/IIIa inhibitor. It has been indicated for unstable angina and myocardial infarction [31]. Compared with another glycoprotein IIb/IIIa inhibitor, abciximab, which is an irreversible antiplatelet, tirofiban is a reversible antiplatelet [31]. Given the relatively long platelet recovery time of abciximab (up to 48 h), hemorrhagic complications are of greater concern for abciximab than for tirofiban (up to 2–4 h) [32]. While another glycoprotein IIB/IIIA inhibitor, eptifibatide, is not available in Korea, the use of tirofiban in ERT has been approved by the Korean Food and Drug Administration for emergency setting.

In the current study, we evaluated revascularization status using AOL and mTICI scale which could assess different dimensions such as recanalization and reperfusion, respectively. The AOL scale can assess directly the performance of MT and can be useful for the estimation of the remnant stenosis because the AOL scale measures the recanalization status at the target occlusive lesion (none, incomplete, complete) [24]. However, it may be possible to ignore the status of the target downstream territory. On the contrary, the mTICI scale estimates the antegrade restoration of the capillary blush so that it could estimate the extent of reperfusion of the target downstream territory [24] and could be advantageous to reflect the thrombogenic events such as thrombus propagation and distal embolization by IST. In this study, we found an important role of tirofiban in reperfusion beyond recanalization. In terms of recanalization, the rate of successful recanalization graded by AOL was the same in both groups (69.5%, respectively). However, the ERT procedure was completed in 81.4% of the patients only after tirofiban was locally injected as a single rescue treatment in the tirofiban group. In addition, the incidence of postprocedural reocclusion on repeat angiographies was much lower in the tirofiban group than in the non-tirofiban group even though the degree of remnant stenosis prior to rescue treatment tended to be higher in the tirofiban group. These findings suggest that tirofiban may stabilize the thrombogenic environment in the stenotic lesion and reduce the use of additional MT strategies. Subsequently, endothelial damage and endovascular procedure time may also be reduced.

Beyond recanalization, the reperfusion status should always be considered. Reperfusion includes restoration of blood flow into the distal branches and the deep brain [24, 33]. In this study, even if both groups had the same rate of successful recanalization, the rate of successful reperfusion was higher in the tirofiban group than in the non-tirofiban group. In most cases, tirofiban infusion was administered immediately after the first partial recanalization in cases with a suspicion of underlying stenosis or in cases of reocclusion after recanalization. Early local tirofiban infusion may contribute to the prevention of downstream embolization by local thrombosis, which may result in a better reperfusion status [34].

Multiple studies have reported that the application of glycoprotein IIb/IIIa inhibitors increases the risk of postprocedural hemorrhagic complications. Although glycoprotein IIb/IIIa inhibitors are not fibrinolytic agents, a high rate of fatal intracerebral hemorrhages has been reported [35, 36]. These studies reported that glycoprotein IIb/IIIa inhibitors were administered intravenously for at least 12 h. A relatively high dose of glycoprotein IIb/IIIa inhibitors may be needed to elicit the appropriate action when it is administered intravenously. In addition, because patients were enrolled up to 2011 in these studies, new MT techniques may have not been incorporated. Further, similar to the failed ERT trials in 2013 [37–39], the rate of successful reperfusions in these studies was relatively low (61.6% in the tirofiban study). Lower rates of successful reperfusion may be related to a greater final infarct volume, which may be more vulnerable to antithrombotic therapy. In contrast, the present results revealed that tirofiban did not increase intracerebral hemorrhages when it was slowly infused via catheter and administered at a low dose following newer MT treatment. Recent studies have demonstrated that primary stent retrieval effectively removed in situ thrombi in ICAS-LVO [8, 40]. On the other hand, serious hemorrhagic complications were more strongly associated with the final infarction volume than with intravenous thrombolysis or local tirofiban infusion shown in the present study. Our results suggest that the appropriate administration of tirofiban may maintain the reperfusion status and reduce the infarct volume. Therefore, the risk of serious hemorrhagic complications may be reduced following tirofiban administration.

This study had several limitations. First, given the retrospective design with a relatively small sample size, data may be skewed, and hidden confounders may have affected the direction of treatment. Additionally, a previous study reported that stenosis length affected treatment outcomes [41]. However, stenosis length could not be measured in the present study because of the interference caused by IST and LVO or vessel injury by primary MT. Nevertheless, our main results are supported by multivariable adjustments, which consisted of well-known predictors. Second, the dose and infusion speed of tirofiban was not prespecified because of the retrospective nature of this study. However, from early experiences and previous anecdotal reports, the dose did not vary extensively. For example, the total amount of tirofiban infusion was low and only varied from 0.5 mg to 2.0 mg among all three stroke centers. Additionally, the infusion speed was between 0.05 and 0.1 mg/min. Third, although patients with LVO and underlying ICAS were included in this study, some patients also had atrial fibrillation whereas the frequency did not differ between groups. These cases may have contaminated the effectiveness and outcomes of local tirofiban infusion on IST of ICAS-LVO. To overcome this limitation, we conducted further analyses that excluded patients with atrial fibrillation (shown in the Supplemental document); however, the clinical and angiographic outcomes did not differ. Finally, old-generation contact aspiration catheters were used in some portions of the primary MT devices. However, the frequency of the use did not differ between the groups in our post-hoc analysis. Considering that the main goal of this study was to identify rescue ERT strategies for underlying ICAS after thrombectomy, the effect of MT devices would be minor.

## Conclusions

Local tirofiban infusion following MT may be a feasible treatment option for patients with ICAS-LVO. The rate of favorable outcomes was higher and the rate of serious hemorrhagic complications was lower in patients who received tirofiban infusion as a rescue treatment than in patients who did not receive infusions of tirofiban as a rescue treatment.

## Supplementary information

**Additional file 1: Table S1.** Comparison of baseline characteristics between the tirofiban and non-tirofiban groups. **Table S2.** Details of endovascular treatment and clinical outcomes. **Table S3.** Binary logistic regression analysis for favorable clinical outcome. **Table S4.** Binary logistic regression analysis for serious hemorrhagic complications.

## Data Availability

The datasets used and/or analyzed during the present study are available from the corresponding author on reasonable request.

## Abbreviations

AIS: Acute ischemic stroke
AOL: Arterial occlusive lesion
ASIAN KR: Acute Stroke due to Intracranial Atherosclerotic occlusion and Neurointervention Korean Retrospective
ASPECTS: Alberta Stroke Program Early CT Scores
ERT: Endovascular revascularization therapy
ICA: Internal carotid artery
ICAS: Intracranial atherosclerotic stenosis
IST: In situ thrombosis
LVO: Large vessel occlusion
MCA: Middle cerebral artery
mRS: Modified Rankin Scale
MT: Mechanical thrombectomy
mTICI: Modified Treatment In Cerebral Ischemia
NIHSS: National Institute of Health Stroke Scale
rtPA: Recombinant tissue plasminogen activator
SAH: Subarachnoid hemorrhage
Tirofiban ASSIST: Tirofiban for Acute Serious Stroke Due to Intracranial in situ Thrombosis
