# Supplementary material for: Local tirofiban infusion for remnant stenosis in large vessel occlusion: tirofiban ASSIST study
Source: BMC Neurol. 2020 Jul 20;20:284. doi: 10.1186/s12883-020-01864-4 (PMC7370431; doi:10.1186/s12883-020-01864-4)
Supplement: Supplementary file 1 — Additional file 1: Table S1. Comparison of baseline characteristics between the tirofiban and non-tirofiban groups. Table S2. Details of endovascular treatment and clinical outcomes. Table S3. Binary logistic regression analysis for favorable clinical outcome. Table S4. Binary logistic regression analysis for serious hemorrhagic complications. [file 12883_2020_1864_MOESM1_ESM.docx]

**SUPPLEMENTAL MATERIAL**

***Supplement tables***

Analyses of the enrolled patients with intracranial atherosclerosis excepting atrial fibrillation.

Table 1. Comparison of baseline characteristics between the tirofiban and non-tirofiban groups

|  | Non-tirofiban Group (n=47) | Tirofiban Group (n=50) | P Value |
| --- | --- | --- | --- |
| Age, median (IQR) | 61 (54-69) | 71 (60.75-76) | 0.005 |
| Female | 10 (21.3%) | 19 (38.0%) | 0.072 |
| Prestroke mRS, median (IQR) | 0 (0-0) | 0 (0-0) | 0.465 |
| Initial NIHSS, median (IQR) | 15 (11-22) | 14.5 (10.75-20) | 0.459 |
| ASPECTS on noncontrast CT, median (IQR) | 8 (5.5-9.5, n=33) | 8 (6-9, n=38) | 0.748 |
| IV-rtPA | 23 (48.9%) | 18 (36.0%) | 0.197 |
| Target occlusion location |  |  | 0.796 |
| Terminal ICA | 6 (12.8%) | 6 (12.0%) |  |
| MCA M1 | 28 (59.6%) | 34 (68.0%) |  |
| MCA M2 | 2 (4.3%) | 1 (2.0%) |  |
| VBA | 11 (23.4%) | 19 (18.0%) |  |
| Hypertension | 30 (63.8%) | 32 (64.0%) | 0.986 |
| Diabetes mellitus | 18 (38.3%) | 15 (30.0%) | 0.389 |
| Dyslipidemia | 21 (44.7%) | 10 (20.0%) | 0.009 |
| Coronary disease | 2 (4.3%) | 4 (8.0%) | >0.678^†^ |
| Smoking | 19 (40.4%) | 18 (36.0%) | 0.654 |
| Prior antiplatelet | 3 (6.4%) | 9 (18.0%) | 0.082 |
| Prior anticoagulant | 1 (2.1%) | 0 | 0.485^†^ |

ASPECTS, Alberta Stroke Program Early CT Score; IQR, interquartile range; ICA, internal carotid artery; MCA, middle cerebral artery; mRS; modified Rankin Scale; NIHSS, National Institute of Health Stroke Scale; rtPA, recombinant tissue plasminogen activator; VBA, vertebro-basilar artery

^†^ Fisher’s exact T test

Table 2. Details of endovascular treatment and clinical outcomes

|  | Non-tirofiban Group (n=47) | Tirofiban Group (n=50) | P Value |
| --- | --- | --- | --- |
| Onset to puncture time | 287 (235-482) | 397 (272-663) | 0.076 |
| Puncture to final angiography time | 83 (60-101) | 63.5 (42-87) | 0.008 |
| Onset to reperfusion time | 381 (312-652) | 484.5 (345-712) | 0.124 |
| First-line endovascular treatment |  |  | 0.124 |
| Aspiration Thrombectomy | 21 (44.7%) | 27 (54.0%) |  |
| Stent retriever | 20 (42.6%) | 22 (44.0%) |  |
| Local fibrinolytics | 1 (2.1%) | 1 (2.0%) |  |
| Angioplasty | 5 (10.6%) | 0 |  |
| Immediate reocclusion after first endovascular treatment | 9 (20.5%) | 22 (44.9%) | 0.013 |
| Degree of residual stenosis prior to rescue treatment (%) | 80 (71.2-85.7) | 83.8 (79.2-90) | 0.021 |
| Rescue treatments |  |  |  |
| Local Tirofiban infusion only | 0 | 42 (84.0%) | <0.001 |
| Stent retrieval to aspiration | 1 (2.1%) | 0 | 0.485^†^ |
| Aspiration to stent retrieval | 7 (14.9%) | 3 (6.0%) | 0.150 |
| Intracranial balloon angioplasty | 9 (19.1%) | 2 (4.0%) | 0.019 |
| Intracranial stenting | 11 (23.4%) | 3 (6.0%) | 0.015 |
| Final AOL 2 or 3 | 32 (68.1%) | 37 (74.0%) | 0.521 |
| Final mTICI 2b or 3 | 31 (66.0%) | 44 (88.0%) | 0.010 |
| Postprocedural reocclusion | 10 (38.5%, n=26) | 1 (2.7%, n=37) | <0.001 |
| Intracranial Hemorrhage |  |  | 0.336 |
| HT type 1 | 3 (6.4%) | 2 (4.0%) |  |
| HT type 2 | 5 (10.6%) | 2 (4.0%) |  |
| PH type 1 | 3 (6.4%) | 1 (2.0%) |  |
| PH type 2 | 5 (10.6%) | 3 (6.0%) |  |
| Subarachnoid hemorrhage | 6 (12.8%) | 0 | 0.011^†^ |
| Intraventricular hemorrhage | 7 (14.9%) | 1 (2.0%) | 0.028^†^ |
| Serious hemorrhagic complication^‡^ | 8 (17.0%) | 3 (6%) | 0.087 |
| Final infarct volume, *ml* (median, IQR) | 38.2 (15.9-119.3) | 20 (8.2-36.9) | 0.021 |
| mRS 0-2 at 3 months | 14 (29.8%) | 26 (52.0%) | 0.026 |
| Mortality | 10 (21.3%) | 4 (8.0%) | 0.063 |

AOL, arterial occlusive lesion; ERT, endovascular revascularization therapy; HT, hemorrhagic transformation; mRS, modified Rankin Scale; MT, mechanical thrombectomy; mTICI, modified treatment in cerebral ischemia; PH, parenchymal hematoma

^†^Fisher’s exact t-test; ^‡^Serious hemorrhagic complications consist of parenchymal hematoma type 2 and/or subarachnoid hemorrhage Fisher grade 3–4.

Table 3. Binary logistic regression analysis for favorable clinical outcome

| Variables | Odds ratio (95% CI) | p value |
| --- | --- | --- |
| Age | 0.909 (0.853-0.968) | 0.003 |
| Female | 0.506 (0.149-1.717) | 0.275 |
| Baseline NIHSS | 0.873 (0.787-0.968) | 0.010 |
| Occlusion location |  | 0.292 |
| Terminal ICA | Ref. |  |
| MCA M1 | 4.058 (0.671-24.561) | 0.127 |
| MCA M2 | 1.948 (0.076-49.995) | 0.687 |
| VBA | 1.485 (0.177-12.458) | 0.716 |
| Onset to puncture time | 1.000 (0.999-1.001) | 0.963 |
| Puncture to final reperfusion time | 0.990 (0.976-1.003) | 0.130 |
| Successful reperfusion | 1.661 (0.420-6.562) | 0.469 |
| Rescue balloon angioplasty and/or stenting | 0.446 (0.098-2.032) | 0.297 |
| Local tirofiban infusion | 3.520 (0.967-12.808) | 0.056 |

ICA, internal carotid artery; MCA, middle cerebral artery; NIHSS, National Institute of Health Stroke Scale; rtPA, recombinant tissue plasminogen activator; VBA, vertebro-basilar artery

^†^Serious hemorrhagic complications consist of parenchymal hematoma type 2 and/or subarachnoid hemorrhage Fisher grade 3–4.

Table 4. Binary logistic regression analysis for serious hemorrhagic complications

| Variables | Odds ratio (95% CI) | p value |
| --- | --- | --- |
| Age | 0.969 (0.892-1.054) | 0.467 |
| Intravenous rtPA | 0.175 (0.012-2.537) | 0.202 |
| Prior use of oral antiplatelet or anticoagulant | 3.348 (0.253-44.275) | 0.359 |
| Onset to final reperfusion time | 0.998 (0.993-1.004) | 0.526 |
| Local tirofiban | 1.190 (0.089-15.910) | 0.896 |
| Final infarct volume | 1.009 (1.000-1.018) | 0.049 |

ERT, endovascular revascularization therapy; rtPA, recombinant tissue plasminogen activator
